# Methylation of the telomerase gene promoter region in umbilical cord blood of patients with gestational diabetes mellitus is associated with decreased telomerase expression levels and shortened telomere length

**DOI:** 10.3389/fendo.2025.1502329

**Published:** 2025-03-11

**Authors:** Shuhua Liu, Liping Xu, Yan Cheng, Dehong Liu, Bin Zhang, Xianxia Chen, Mingming Zheng

**Affiliations:** ^1^ Department of Obstetrics and Gynecology, Hefei Maternal and Child Health Hospital, Hefei, China; ^2^ Department of Obstetrics and Gynecology, Anhui Women and Children’s Medical Center, Hefei, China; ^3^ Department of Obstetrics and Gynecology, Maternal and Child Medical Center of Anhui Medical University, Hefei, China; ^4^ Fifth School of Clinical Medicine, Anhui Medical University, Hefei, China

**Keywords:** gestational diabetes mellitus, telomere length, telomerase, TERT, TERC, methylation

## Abstract

**Objective:**

This study speculates that gestational diabetes mellitus (GDM) may reduce fetal telomere length (TL),which may be related to modification of methylation in the promoter region of the telomerase (TE) gene promoter region.

**Methods:**

In this study, umbilical cord blood samples from patients with and without GDM (N = 100 each) were analyzed by prospective case-control. The TL, TE expression levels, and methylation levels of TERT and TERC gene promoter regions in two groups were measured. The significance of the methylation level of each CpG locus employed logistic regression analysis of R software, and the analysis of covariance (ANCOVA) was used to control the influence of confounding factors. Correlation analysis was performed by the Spearman.

**Results:**

The TL and TE expression levels of the offspring of GDM patients were decreased despite adjusting for PBMI, PWG, and TG. A total of two CpG islands were screened in the promoter region of the TERT gene and three fragments (TERT_2, TERT_3, and TERT_4) containing a total of 70 CpG sites were designed. Additionally, four CpG sites of the TERT gene in the GDM group (TERT_2_40, TERT_2_47, TERT_3_46, and TERT_3_212) showed increased methylation levels compared with the control group (all P < 0.05). In the promoter region of the TERC gene, one CpG island containing 19 CpG loci was screened and designed, and the methylation levels of the two CpG sites were significantly different in TERC_1_67 (0.65 ± 0.21 versus 0.57 ± 0.30; P = 0.040) and TERC_1_120 (0.68 ± 0.23 versus 0.59 ± 0.27; P = 0.014). The methylation levels of TERC gene fragments of GDM patients were significantly higher than those of the control group (0.69 ± 0.06 versus 0.65 ± 0.08, P = 0.001).

**Conclusion:**

This study revealed that GDM may induce decreased TE expression by increasing the methylation levels of TE genes promoter region, thereby reducing the TL.

## Background

Gestational diabetes mellitus (GDM) is the most common metabolic imbalance during pregnancy and has a profound impact on the short- and long-term health of the fetus. Studies suggest that an impaired intrauterine environment is detrimental to an individual’s lifelong health and may significantly increase the risk of diseases such as obesity, diabetes, cardiovascular disease, renal dysfunction, and early-onset metabolic syndrome (1–7). However, the specific mechanism underlying this effect is not fully elucidated. Therefore, current research is actively exploring the association between metabolic pathways and acquired and genetic factors, aiming to unfold novel perspectives in treating these diseases (8–10). Consequently, genome-wide association studies (GWAS) have identified several genetic markers such as single nucleotide polymorphisms (SNPs) and copy number variations (CNVs) that are strongly associated with metabolic disease risk (11–14); however, these genetic variants account only for a small part of an individual’s disease susceptibility. Nevertheless, epigenetic mechanisms may unravel the mystery of genetic deletion of complex phenotypes (15, 16). Studies have shown that organisms are particularly sensitive to environmental stresses and lifestyle choices at specific life stages, such as before and after conception, embryonic development, pregnancy progression, and early childhood. Collectively, these factors predetermine the response patterns of individuals to disease at subsequent life stages (17, 18). Therefore, epigenetic modification during pregnancy has emerged as a means to explore the relationship between environmental effects and fetal metabolic programming (19). Numerous studies have demonstrated that epigenetic changes are associated with adverse health conditions such as maternal malnutrition, obesity, and GDM-exposed offspring (20–23).

Epigenetics explores the mechanism of heritable variation in gene expression without changes in DNA sequence. This imparts diverse gene expression patterns and biological functions to cells and tissues sharing the same genetic sequence. Notably, DNA methylation is an early recognized and widely studied phenomenon in epigenetics (24). It involves the conversion of cytosine to 5-methylcytosine, an important component of the DNA base sequence that co-exists with A, C, T, and G, and is commonly observed in the CpG nucleotide sequence of mammalian DNA (25). Reportedly, an unfavorable intrauterine environment can trigger epigenetic changes, such as differences in methylation at CpG loci and deletion of imprinted genes (26–28). Such changes have a profound impact on chromatin structure and tissue level in many biological processes (29). Moreover, epigenetic variations are potentially transmitted across paternal and maternal generations. Since DNA methylation is a common epigenetic mechanism, its enrichment in CpG islands, especially in gene promoter regions, is often associated with gene missing expression AND repression (30).

Telomeres are repetitive DNA sequences at the ends of chromosomes to prevent their fusion and degradation. According to recent studies, abnormal changes in telomere length (TL) are associated with a wide range of health problems, ranging from glucose metabolism-related disorders, dyslipidemia, arteriosclerosis, obesity, diabetes, and cardiovascular diseases, to various cancer types (31). GDM, a state of impaired glucose metabolism during pregnancy, adversely affects the mother and the growth and development of the fetus and neonates, as well as the cellular structure and function. Previous studies (32, 33) have investigated the association between GDM and telomere changes in progeny; however, the limitations of detection techniques, small sample size, and lack of timely adjustment of confounding factors failed to report any definitive conclusions.

Telomeres are progressively shortened during cell division. This process is affected by various factors including the expression of TE, a key regulator of TL. Specifically, TE uses its TERC, to associate with human telomere-associated protein 1 (hTP1), and utilizes TERT to synthesize telomeric DNA, thereby maintaining the TL and integrity (34, 35). The non-coding RNA molecule (451 nt) at the core of TERC exhibits a highly conserved secondary structure and its sequence variation can affect TE activity significantly. Reportedly, a rat model lacking the TERC gene (TERC−/−G4) revealed that TE loss and consequent telomere shortening promoted growth arrest and aging of pancreatic β cells. In addition, the methylation level of the TERT gene affects its transcriptional activity, thereby indirectly regulating TE activity (36). Precisely, the TERT gene is located at 5p15.33 on chromosome 5 and is about 35,000 bp long containing 16 exons and 15 introns (37),and normal expression of telomerase genes plays a key role in telomerase activity (38).

In the past, it was thought that GDM has a certain impact on the short-term and long-term existence of offspring, but the specific reasons are still uncertain. The purpose of this study was to explore whether there are some changes in telomere epigenetics, i.e., whether there are changes in TL/TE biology and the degree of methylation modification of TE-related genes in the offspring of GDM patients, so as to pave the way for further research and demonstration. It may provide a new epigenetic perspective on the “fetal origin of adult disease” hypothesis.

## Materials and methods

### Study participants

In this prospective cohort study, we collected basic information from the pregnant women (including their demographic and clinical characteristics, as well as follow-up pregnancy progression and outcomes) from November 2022 to October 2023 at the Hefei Maternal and Child Health Hospital, Anhui, China. The research protocol was approved by the Ethics Review Committee of Hefei Maternal and Child Health Hospital, and the participants were enrolled after obtaining written informed consent.

The inclusion criteria were as follows: singleton pregnancy, ≥ 18 weeks, regular prenatal care and delivery, oral glucose tolerance test (OGTT with 75 g of glucose) conducted between 24–28 weeks of gestation. The exclusion criteria were as follows: multiple pregnancies; history of adverse pregnancy; pregnant women with a history of drug use, malignancy, hypertension, type 1 or type 2 diabetes, long-term use of drugs affecting blood glucose, etc., and pregnant women with incomplete medical records.

Moreover, fasting plasma glucose (OGTT-FPG) levels ≥ 5.1 mmol/L, 1-hour blood glucose level (OGTT-1) ≥ 10.0 mmol/L, or 2-hour blood glucose level (OGTT-2) ≥ 8.5 mmol/L were considered diagnostic criteria for GDM.

### Specimen collection

After placental delivery, the cord blood sample was collected in an EDTA anticoagulant tube, followed by centrifugation at 3,000 rpm for 10 min. The isolated plasma and blood cells were then transferred into 2 mL tubes and stored at −80°C. Later, total DNA was extracted from monocytes using ATL lysis buffer (Qiagen, Inc., Valencia, CA), proteinase K, and RNase A buffers (Qiagen, Inc., Valencia, CA) for phenol-chloroform extraction and ethanol precipitation.

### Determination of TL

TL was measured using quantitative PCR as proposed by Cawthon (39, 40). The reference gene was selected as a single copy of 36B4 as reported previously (39, 40). Amplification was performed using suitable primers (40) on an ABI 7900 real-time PCR instrument (Applied Biosystems, Foster City, California) by optimization using the melting curve program. The telomere gene primer sequences were as follows: tel F1: 5′- CGGTTTGTTTGGGTGGGTTTGGGTTTGGGTTTGGGTTTGGGTTTGGGTTTGGGTT- 3′; tel R1: 5′- GGCTTGCCTTACCCTTACCCTTACCCTTACCCTT- 3′. The internal control gene primers were as follows: 36B4-F: 5′- CAGCAAGTGGGAAGGTAATCC- 3′; and 36B4-R: 5′- CCCATTCTATCATCAACGGGTACAAAA- 3′. The quantitative PCR steps were as follows: 95°C for 1 min; 40 cycles at 95°C for 5 sec and 55°C for 45 sec. The relative TL was calculated as follows: TL (T/S) = 2^−ΔΔCt^, where ΔCt = mean Ct (telomere) − mean Ct (internal reference gene) and ΔΔCt = ΔCt (sample to be measured) − ΔCt (corrected sample).

The TE expression level and oxidative stress indicators (SOD, 8-OHdG, and MDA) were determined by using an ELISA kit (ELISA, China). The absorbance at 450 nm was recorded using a microplate reader to calculate the amount of protein of interest in the sample using a standard curve.

### DNA extraction and bisulfite modification

The DNA from umbilical cord blood samples collected from GDM patients and healthy controls was isolated using the QIAamp DNA Blood MiniKit (Qiagen,Inc.,Germany). Thereafter, DNA (400 ng) was treated with sodium bisulfite using the EZ DNA Methylation-Gold Kit (Zymo Research) according to the manufacturer’s instructions. Later, DNA methylation was analyzed by MethylTarget^®^ (Genesky Biotechnologies Inc.), an NGS-based method enabling simultaneous sequencing of multiple specific CpG islands to calculate methylation levels at each CpG site with high-depth sequencing data.

### Multiplex PCR amplification

For DNA methylation detection, two regions of TERC and TERT were sequenced using high-quality sequencing primers as follows: Primer F = Illumina adapter sequence 1 + specific amplification Forward primer; Primer R = Illumina adapter sequence 2 + specific amplification Reverse primer. The target region amplification of transformed samples employed multiplex PCR amplification using optimized primer panels designed from bisulfite-converted DNA using methylation primer software. The PCR reaction (final volume 20 μL) consisted of 1 × reaction buffer (Takara) containing 3 mMMg2+, 0.2 mM dNTPs, 0.1 μM of each primer, 1 U of HotStarTaq polymerase (Takara), and 2 μL of template DNA. The amplification steps were as follows: An initial denaturation for 2 min at 95°C, 20 sec at 94°C, 11 cycles of 40 sec at 63°C with a temperature reduction step of 0.5°C per cycle, 1 min at 72°C, followed by 24 cycles of 20 sec at 94°C and 30 sec at 65°C. The primer details are listed in **Table 1**.

**Table 1:** Primer information at the methylation level of TERC and TERT genes.

Gene fragments	Position (hg38)	Length	Forward Sequence(5′-3′)	Reverse Sequence(5′-3′)
TERC_1	chr3:169764724-169764499	226	GYGAGGGYGAGGTTTAGGTTTTT	ACAACACACTAACCCAATCAATCAA
TERT_2	chr5:1295504-1295738	235	GGAGATTTAGGGTTGTTTTTAGGTT	CCRCCTAAAAACCTACAAAAAAAAATAAC
TERT_3	chr5:1293872-1294141	270	TTTGTYGTTTGAGGAGTAGAGGA	ACRCCCRTTAAACAAAAATCCTAAAC
TERT_4	chr5:1294301-1294553	253	TTTTYGGGGTTTATTAGYGTGTG	CCCCRAAACCTTCACCAC

### Bioinformatics analysis

The quantification of the molar concentration of the library was followed by obtaining FastQ data using high-throughput sequencing in 2 × 150 bp paired-end sequencing mode on the Illumina Nova seq platform. In addition, sequencing data of samples revealed that the efficiency of base C to T conversion was increased after bisulfite treatment. The level of methylation modification at each CpG site in quantitative data was determined by dividing the number of reads methylated (i.e., the number of reads where base C was detected) by the total number of reads at a site. According to the grouping data, the logistic regression analysis of R software. The methylation modification level of a quantified fragment was the average of methylation levels at all CpG sites on the fragment. According to the group data, R software logistic regression analysis was used to identify the fragments with significant differences in methylation level, and P < 0.05 was considered statistically significant.

### Statistical methods

SPSS 26.0 software (IBM, New York, USA) was used for statistical analysis. For variables showing normal or near-normal distribution, the homogeneity test of normality and variance was performed and resultant values were expressed as a mean ± standard deviation. In the absence of normal distribution, median and interquartile ranges were used to describe the tendency of the data to concentrate or disperse. Variables with normal distribution and homogeneity were subjected to T-test independently. Conversely, the Mann-Whitney U test was used for comparison between the groups, and analysis of covariance (ANCOVA) was used to control confounders. The result visualization utilized R software (R-4.3.2, UoA, New Zealand). Moreover, Spearman was used to analyze the correlation between TL and clinical indicators. P < 0.05 were considered statistically significant.

## Results

### Comparison of demographic and clinical data between GDM patients and healthy individuals

The results revealed no difference in age between the two groups (P > 0.05; **Table 2**); however, the BMI, PWG, BG, OGTT-FPG, OGTT-1, OGTT-2, and TG levels in patients with GDM were higher than those in healthy individuals (all P < 0.05), which coincided with the pronounced disease features of GDM.

**Table 2:** Demographic information and clinical indicators of GDM group an control group.

Variables	GDM group (N=100)	Control group (N=100)	*P value*
Maternal age (years)	30.25±3.64	30.38±3.94	0.075
PBMI (kg/m^2^)	23.62±3.25	21.36±2.32	<0.001
PWG (kg)	14.51±5.21	12.25±5.42	0.035
BG (mmol/1)	4.67±0.46	4.21±0.31	<0.001
OGTT-FPG (mmol/1)	5.36±0.36	4.51±0.24	<0.001
OGTT_1 (mmol/1)	9.38±1.75	7.26±1.32	<0.001
OGTT_2(mmol/1)	7.86±1.51	6.21±1.13	<0.001
GA (weeks)	39.22±0.76	39.68±0.89	0.013
TG (mmol/1)	3.32±1.31	2.74±1.35	0.026
BW (g)	3481.09±365.82	3473.76±426.15	0.435
Newborn gender: Boy/Girl	52/48	54/46	0.887
8-OHdG (pg/mL)	216.21±56.75	224.54±52.68	0.231
MDA (ng/mL)	6.54±1.61	7.01±1.59	0.285
SOD (nmol/mL)	12.56±3.58	12.71±3.64	0.886
TE (ng/mL)	17.35±1.48	11.36±1.76	<0.001

GDM, Gestational diabetes mellitus; PBMI, Pre-pregnancy body mass index; PWG, Pregnancy weight gain; OGTT-FPG, fasting blood glucose; OGTT_1, Oral glucose tolerance test 2 hour blood glucose; OGTT_2, Oral glucose tolerance test 2 hour blood glucose; TG, Triglyceride; BW, Birth weight; GA, Gestational age; 8-OHdG, 8-hydroxy-2' -deoxyguanosine; MDA, Malondialdehyd; SOD, Superoxide dismutase; TE, Telomerase.

### Comparison of TL in umbilical cord blood of patients with and without GDM

**Figure 1:**
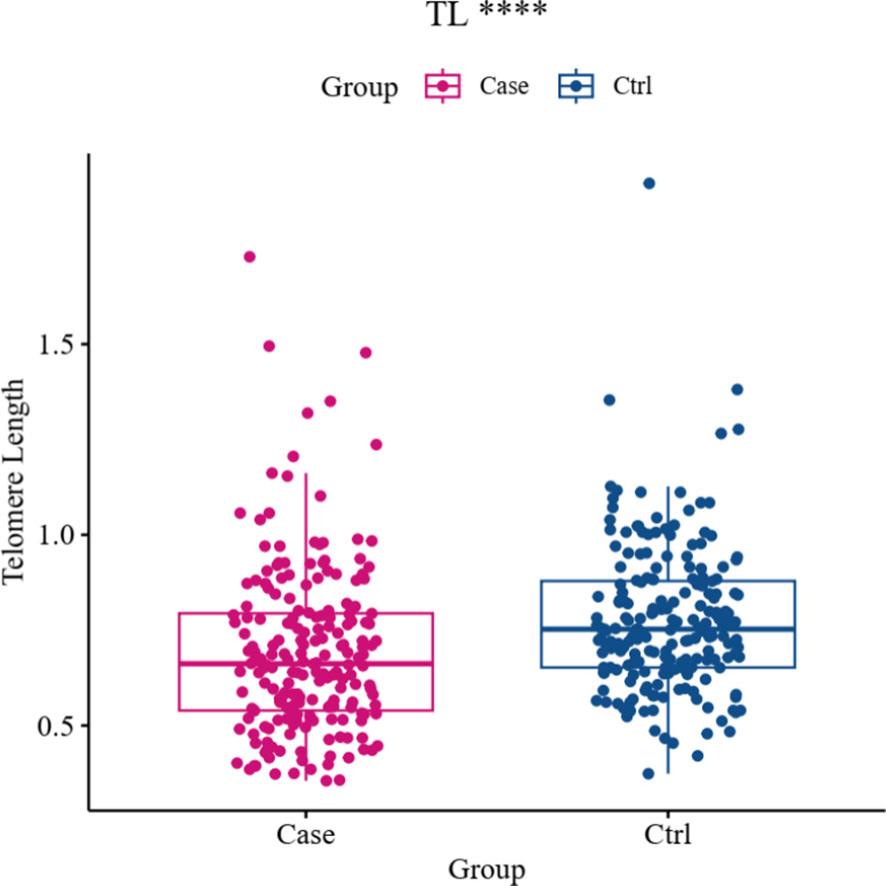
Comparison of TL in umbilical cord blood between the GDM group and the Ctrl group (**** for <0.001,Ctrl for control).

As shown in **Figure 1**, the TL of the offspring of GDM patients was significantly shorter than that of healthy individuals (0.62 ± 0.31 versus 0.75 ± 0.21, P < 0.001).

### Correlation analysis between TE expression levels, TL, and clinical indicators

**Figure 2:**
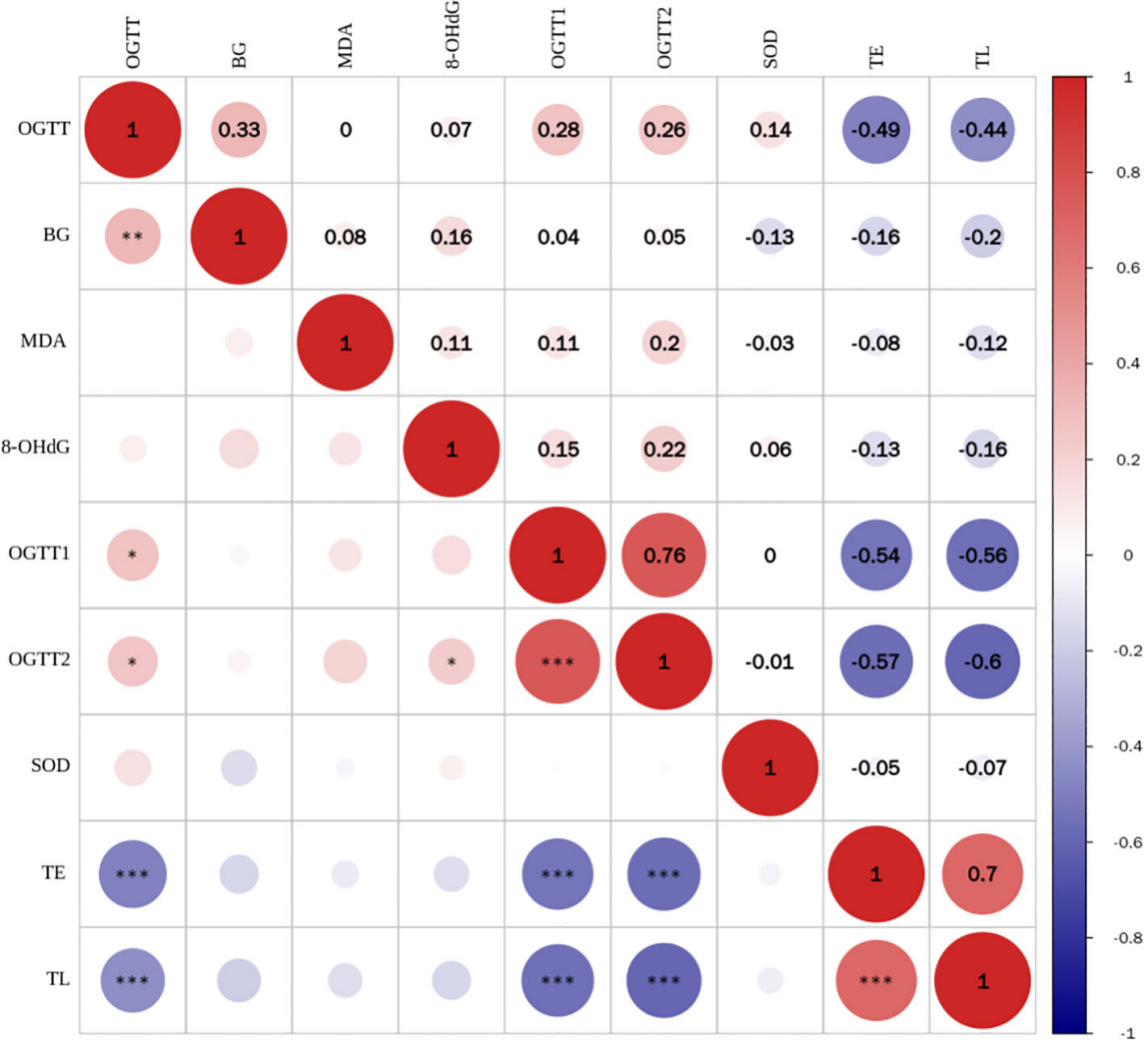
Correlation analysis between TL and telomerase expression levels and blood glucose and oxidative stress indicators (*** for <0.001).

As shown in **Figure 2**, TE expression level was positively correlated with TL (r = 0.699, P < 0.001), and negatively correlated with blood glucose indicators OGTT-FPG (r = -0.487, P < 0.001),OGTT-1h (r = -0.539, P < 0.001) and OGTT-2h (r = -0.568, P < 0.001). TL was negatively correlated with blood glucose: OGTT-FPG (r = -0.435, P < 0.001), OGTT-1h (r = -0.558, P < 0.001) and OGTT-2h (r = -0.597, P 0.001). However, no correlation was observed between TE expression level and TL and BG, MDA, SOD, 8-OHdG (all P > 0.05).

### Comparison of methylation levels of the TERT and TERC gene promoter region between the GDM and control groups

As shown in **Table 3**, two CpG islands were screened in the promoter region of the TERT gene, and three fragments (TERT_2, TERT_3, and TERT_4) were designed to analyze DNA methylation levels. Results revealed that the DNA methylation levels of 70 CpG loci were different in the umbilical cord blood samples of the offspring of GDM patients relative to those of healthy individuals. However, One CpG island was screened in the promoter region of TERC gene as per the screening criteria, and one fragment containing 19 CpG sites was designed for the TERC gene.

**Table 3:** Genome detection information of CpG islands in the promoter region of TERT and TERC gene.

Gene	CpG island location	CpG island length(bp)	Gene fragments	Number of covered CpG sites	Distance from TSS(bp)
TERT	Chr5:1295487-1295736	250	TERT_2	24	436
	Chr5:1293880-1295468	1589	TERT_3	21	927
			TERT_4	25	515
TERC	Chr3:169764529-169765309	781	TERC_1	19	249

### Comparison of CpG locus of the TERT and TERC gene promoter regio of umbilical cord blood mononuclear cells of GDM and control groups

**Figure 3:**
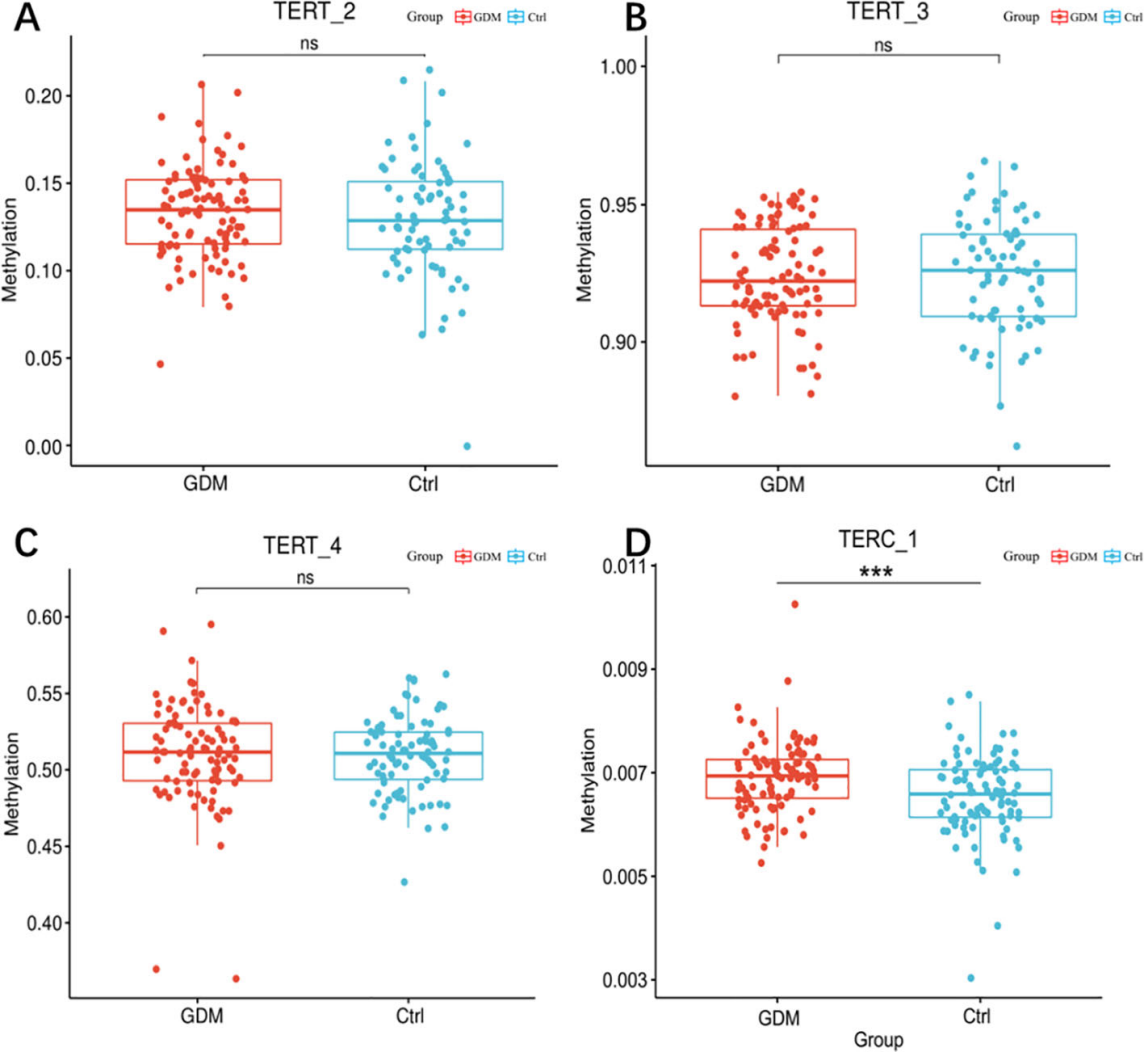
Methylation levels of hTERT and TERC gene promoter regions in cord blood of GDM patients and healthy controls. Note: Methylation levels of TERT and TERC gene promoter fragments in cord blood of GDM patients and healthy controls. **(A)** box scatter plot of tert_2fragment methylation levels; **(B)** Box scatter plot of tert_3 fragment methylation level; **(C)** Box scatter plot of tert_4 fragment methylation level; **(D)** Box scatter plot of terc_1 fragment methylation levels. Student t test was used for comparison between groups. ns for *P* 0.05, *** for *P* = 0.001.

As shown in **Figures 3A–C**, among the 70 CpG loci, four CpG loci in the GDM group showed increased methylation levels compared with the control group as follows: TERT_2_40 (3.39 ± 2.19 versus 2.11 ± 2.59, P = 0.001), TERT_2_47 (6.87 ± 3.36 versus 5.49 ± 5.27, P = 0.039), and TERT_3_46 (86.83 ± 6.31 versus 85.23 ± 3.36, P = 0.037), and TERT_3_212 (98.18 ± 0.99 versus 98.67 ± 1.87, P = 0.034).As shown in **Figure 3D**, there were two Cpg loci in the promoter region of TERC gene in the cord blood of GMD patients, TERC_1_67 (0.65 ± 0.21 versus 0.57 ± 0.30, P = 0.040) and TERC_1_120 (0.68 ± 0.23 versus 0.59 ± 0.27, P = 0.014), which were significantly higher than those in the control group.

### Comparison of methylation levels of the TERT and TERC gene promoter region fragments in patients with and without GDM

**Figure 4:**
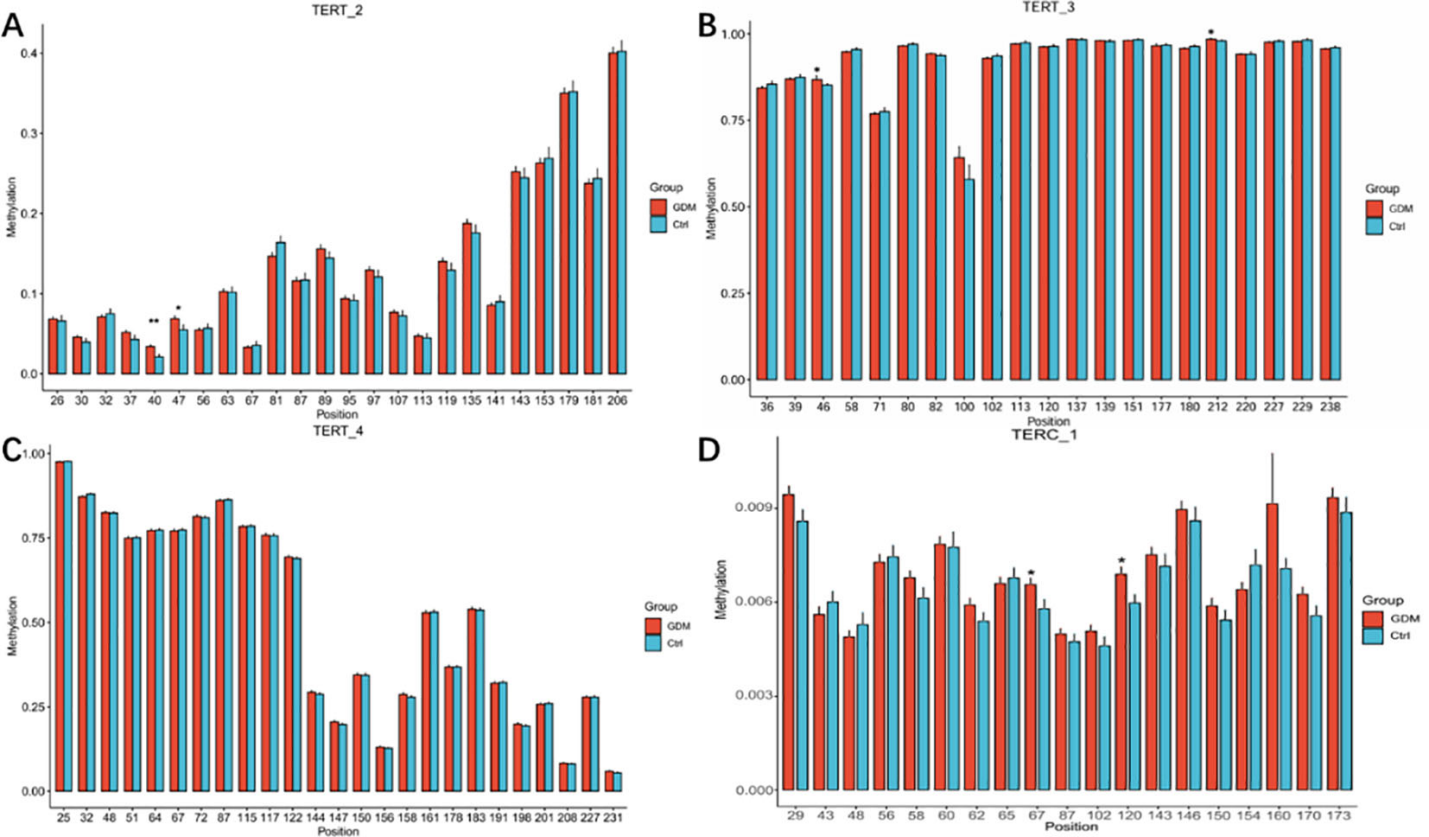
Methylation levels of CpG loci in the promoter region of TERT and TERC genes in cord blood between GDM group and control group Note: methylation levels of CpG sites in the promoter region of TERT and TERC genes in cord blood of GDM group and control group. **(A)** Histogram of methylation levels of all CpG sites on TERT 2 fragment; **(B)** Histogram of methylation levels of all CpG sites on TERT 3 fragment; **(C)** Histogram of methylation levels of all CpG sites on TERT_4 fragment; **(D)** Histogram of methylation levels of all CpG sites on TERC_1 fragment. The abscissa in the bar graph represents the CPG site on each fragment, and the ordinate represents the methylation level (range 0-1).Student t test was used for comparison between groups.* for *P* < 0.05,** for *P* < 0.01.

The mean of methylation levels of all CpG sites on each fragment was used as the methylation level of that fragment and used to compare the methylation levels of GDM and the control groups. As shown in **Figures 4A–C**, the TERT_2 (13.38 ± 2.63 versus 12.98 ± 3.39, P = 0.380) and TERT_4 (51.07 ± 3.40 versus 50.96 ± 2.49, P = 0.808) fragments in the GDM group showed increased methylation, however, the difference was non-significant for TERT_3 (92.39 ± 1.79 versus 92.46 ± 2.06, P = 0.795).As shown in **Figure 4D**, the methylation level of the offspring in the TERC_1 GDM group was significantly higher than that in the control group (0.69 ± 0.06 *vs*. 0.65 ± 0.08, P = 0.001).

## Discussion

In the present study, we re-evaluated the variation in relative TL in the cord blood samples of patients with GDM. Our results revealed that the offspring of GDM patients showed pathophysiological characteristics of significantly reduced TL and TE activity compared with healthy individuals. In addition, correlation analysis showed a positive correlation between TE and TL, both of which were negatively correlated with blood glucose levels. Considering the regulatory role of TE activity in maintaining TL in mammals, and the effect of the methylation level of the TE promoter region on TE expression (41), we used bisulfite sequencing technology for an in-depth analysis of methylation of the TE gene promoter region in cord blood mononuclear cells of the offspring of GDM patients to find indirect evidence of TE expression inhibition. The results showed that the methylation levels were increased at two specific CpG loci in the TERC gene promoter region and the whole gene fragment of TERC. Similarly, four of the 70 CpG loci in the TERT gene showed increased methylation in the neonatal umbilical cord blood mononuclear cells of pregnant women with GDM. According to previous studies, hypermethylation is often associated with decreased gene transcriptional activity (42–45). Collectively, these findings may suggest an epigenetic pathological mechanism that leads to the shortening of TL in the umbilical cord blood of GDM patients by increasing the degree of methylation of the TE gene, thereby decreasing TE activity.

The methylation level of the TERT promoter region plays a key role in cell carcinogenesis. In most cases, the hyper-methylated state of the TERT promoter region is associated with attenuation of TE activity and transcriptional expression of TERT mRNA. Similarly, previous studies have demonstrated that increased methylation at CpG loci causes down-regulation of gene activity. However, there are exceptions to the TERT promoter, where the partially methylated form is not expressed differently from TE activity (42). Notably, hypermethylation of the TERT promoter has been strongly associated with gastric (46), cervical and ovarian cancers (41). The modification of DNA methylation within cancer cells is often characterized by hypomethylation of CpG sites and hypermethylation of specific CpG islands, leading to the silencing of tumor suppressor genes such as p16 and hMLH1 (as part of DNA mismatch repair mechanisms) (44, 45), which may be related to differences in TERT methylation status in different cell lines.

The activity of TE is limited by its functional properties and is silent in most adult human cells, but not in those cell types with high proliferative potential, such as embryonic stem cells, germ cells, rapidly dividing somatic cells, and tumor cells (47).Umbilical cord blood mononuclear cells are mononuclear cells in umbilical cord blood, mainly including lymphocytes and monocytes. The changes of telomere and telomerase in them can more faithfully reflect the state of primitive stem cells in early human development than in fetal peripheral blood. And studies suggest that umbilical cord blood mesenchymal cells can represent the role of fetal tissue in function (48). Our citation notes for discussion modifications and references.

There are many factors that lead to telomere biological changes. Although PBMI, PWG, BG and other factors have been adjusted in this study, there are also common factors such as diet, stress and other factors that need to be taken into account. Because pregnant women have many subjective factors, these factors are difficult to quantify, so they were not included in the study. Although changes in telomere length and telomerase gene methylation have been observed, whether these changes are associated with adult onset diseases still needs further follow-up research. In addition, in addition to methylation factors, whether epigenetic changes such as histone modification and acetylation are related to telomere shortening still needs to be explored. The sample size of this study is limited and it is a local population sample. Whether there are the same problems in other regions or ethnic groups still needs to expand the sample size and more regional populations to be included in the study. However, this study also has certain advantages. Although our sample size is limited, we detected telomere changes, which is enough to show that the adverse maternal environment may lead to epigenetic changes, and may also be the basis for the pathogenesis of related diseases in the future. Exploring the changes in telomere biological characteristics may open up a novel research path to clarify the adverse consequences of maternal GDM environment on offspring health.

In this study, we not only observed the direct effects of the GDM-induced hyperglycemic environment on the telomeric system of the progeny, but also demonstrated epigenetic changes in the TE promoter region, which we speculate may lead to telomeric system alterations. We will continue to study these speculations in the future.

## Data Availability

The original contributions presented in the study are included in the article/supplementary material. Further inquiries can be directed to the corresponding authors.
